# Treatment of non-sterile biogas slurry from a pig farm using microalgae isolated from the activated sludge of sewage plants

**DOI:** 10.1128/spectrum.00688-25

**Published:** 2025-09-09

**Authors:** Yisong Wei, Yinghuan Chen, Tianzhen Zhao, Xu Zhang, Ning’ao Wang, Leqiong Shen, Ying Liang, Shaomei Ye, Hongzhi He

**Affiliations:** 1 Guangdong Laboratory for Lingnan Modern Agriculture, College of Natural Resources and Environment, South China Agricultural University 214165, Guangzhou, China; 2 Guangdong Provincial Key Laboratory of Agricultural & Rural Pollution Abatement and Environmental Safety, College of Natural Resources and Environment, South China Agricultural University 214165, Guangzhou, China; 3 Guangdong Engineering Technology Research Centre of Modern Eco-agriculture and Circular Agriculture, College of Natural Resources and Environment, South China Agricultural University 214165, Guangzhou, China; 4 Key Laboratory of Agro-Environment in the Tropics, Ministry of Agriculture and Rural Affairs, College of Natural Resources and Environment, South China Agricultural University 214165, Guangzhou, China; 5 Zhongshan Agricultural Product Quality and Safety Inspection Institute, Zhongshan, China; Swansea University, Swansea, United Kingdom

**Keywords:** biogas slurry, microalgae, microbial composition, metagenome, antibiotic resistance genes

## Abstract

Microalgae treatment is regarded as a green and environmentally acceptable method of treating pig farm biogas slurry (BS). Numerous studies have been conducted on the use of microalgae to treat sterilized BS. Nevertheless, in large-scale application settings, this method will undoubtedly result in high costs and low efficiency. In this study, microalgae were isolated from activated sludge, and their capacity to treat non-sterile BS with high ammonium nitrogen levels was investigated, along with examining alterations in microbial composition within BS. The results showed that both *Chlorella* sp. HH01 and *Chlorella* sp. HS02 treatments effectively removed ammonia nitrogen and phosphorus from BS. They also showed some removal capabilities for chemical oxygen demand, heavy metals, and antibiotics. Algal treatment significantly reduced the number of species, richness, variety, and pathogens within the microorganisms of BS, according to the results of metagenomic research. Meanwhile, algal treatment demonstrated a significant removal effect on certain antibiotic resistance genes, while increasing the abundance of some others. This study provides a comprehensive exploration of microalgae-mediated mitigation effects on antibiotic resistance genes in BS.

**IMPORTANCE:**

Numerous studies have been conducted on the use of microalgae to treat sterile biogas slurry (BS). However, in large-scale applications, this approach undoubtedly results in high costs and inefficiencies. Therefore, it is crucial to identify microalgae capable of growing in non-sterilized and undiluted BS while effectively treating major pollutants. The findings of this study reveal that microalgae isolated and purified from activated sludge in sewage treatment plants can withstand crude BS containing high concentrations of ammonia nitrogen and effectively remove ammonia nitrogen and total phosphorus. Additionally, they exhibit some removal capabilities for chemical oxygen demand, heavy metals, pathogens, antibiotics, and certain antibiotic resistance genes.

## INTRODUCTION

China’s demand for meat products has been steadily rising in recent years due to the country’s economic development, which has accelerated the growth of animal husbandry. However, it has also resulted in significant pollution issues (1, 2). According to data from the Ministry of Agriculture and Rural Affairs of China, the production of breeding pigs in 2022 was approximately 1.78 billion. Consequently, a significant amount of pig farm waste was produced, including pig manure, pig urine, and flushing water, which contained many harmful substances (1, 3). Nowadays, most pig farms treat their manure using anaerobic digestion, which produces biogas for energy recovery while eliminating most organic debris and dangerous germs (3).

However, considerable amounts of pollutants like N, P, heavy metals, antibiotics, and pathogens are still present in the substantial volume of biogas slurry (BS) produced following anaerobic digestion (3). It can seriously endanger both human health and the ecological environment if improperly handled and released into the environment (1). Furthermore, applying conventional methods to remove these contaminants from pig farm BS is challenging (2). The high N and P removal rates of microalgae-based wastewater treatment have attracted interest because they are more cost-effective, efficient, and have a reduced carbon footprint compared to previous approaches (4). As a result, treating pig farm BS with microalgae may result in carbon capture, biomass production, and nitrogen recovery while also drastically lowering greenhouse gas emissions during the treatment process (1). The process of converting wastewater nutrients into useful biomass has garnered a lot of interest. For instance, algal biomass with a high lipid content can be used to produce bioenergy, while deoiled algae waste can be utilized as a biofertilizer (5).

Numerous studies have examined the use of microalgae to treat pig farm BS; however, most of these studies call for several pretreatments, including microbial inactivation, dilution, and sterilization, to minimize microalgae toxicity and accomplish adequate pollutant removal (2, 6). Nevertheless, these techniques are costly, time-consuming, and energy-intensive. The use of microalgae to treat sterilized BS has been the subject of numerous investigations (2, 7). However, there is not much research on using unsterilized pig farm BS to cultivate microalgae (8, 9). Thus, it is critical to create an economical way to treat unsterilized raw BS directly (10).

The objectives of this study are to separate and purify microalgae from activated sludge in sewage treatment facilities, assess how well they can withstand and remove pollutants from unsterilized raw BS from pig farms, and examine the microbiological makeup and functional genes of BS throughout the treatment process.

## MATERIALS AND METHODS

### Microalgae and pig farm biogas slurry sources

The two strains of microalgae, *Chlorella* sp. HH01 (PQ357523) and *Chlorella* sp. HS02 (PQ824500), employed in this investigation were isolated from the activated sludge of Hanhai Wastewater Plant and the Second Wastewater Plant in Heshan City, Guangdong Province, respectively. Our group has already published a report on *Chlorella* sp. HH01 (7). The 18S rRNA Blast results for the two microalgal strains are displayed in Table S1. The purified microalgal strains were cultured in Erlenmeyer flasks containing BG11 medium at 28°C ± 0.5°C, 10,000 lux of light, and a light-dark cycle of 12 h:12 h; the bottles were manually shaken three to four times a day. The BS was extracted from a large-scale pig farm in Yunfu City, Guangdong Province. To get rid of any suspended particles, it was brought back to the lab and filtered through three layers of filter paper with a pore size of 80–120 μm. The properties of BS were NH_4_^+^-N, 598 mg L^−1^; TP, 22 mg L^−1^; COD, 967 mg L^−1^; and pH 8.4.

### Design of experiments and measurement of indicators

The experiment was carried out in 500 mL conical flasks, each holding 240 mL of non-sterile BS. Bottles inoculated with two distinct microalgae (*Chlorella* sp. HH01 and *Chlorella* sp. HS02) were assigned to HH01 and HS02 treatments, respectively. CK is defined as the control group of biogas slurry without microalgae treatment, while CK0 is the same as CK on day 0 with three replicates for each treatment.

The algal cells obtained by centrifugation were inoculated to reach an initial OD_680_ in algal treatments of 0.7, as the OD_680_ of CK0 was 0.6. The culture conditions were identical to those outlined in “Microalgae and pig farm biogas slurry sources.” To assess OD_680_, pigment concentration, chlorophyll fluorescence, water quality, and pH, samples were collected on days 0, 2, 4, 6, and 8 of the experiment. On day 8, algal cells were gathered for metagenomic investigation, elemental determination, and the detection of dry weight and lipid content.

Using 6 mL of algal solution, algal cells were collected by centrifugation to determine the pigments. Algal cells were then combined with 6 mL ice-cold 80% acetone and extracted for 24 h at 4°C. The solution was then centrifuged for 10 min at 6,000 rpm, and the absorbance values of the supernatant at 663, 646, and 470 nm were measured. The formula outlined by Lichtenthaler and Wellburn (11) was used to determine the amounts of carotenoids (Cars), chlorophyll *a* (Chl *a*), and chlorophyll *b* (Chl *b*). After being pretreated in the dark for 20 min, the sample’s OJIP curve was measured using a chlorophyll fluorescence analyzer (AquaPen AP-P 100), and the data were analyzed using FluorPen software. The pH value was determined using the MP511 laboratory portable pH meter. The concentration of NH_4_^+^-N, TP, and COD was determined according to the standards HJ 535-2009, GB 11893-1989, and HJ 828-2017 of China, respectively.

Following 8 days of incubation, the biomass was separated by centrifugation (Eppendorf 5804R) at 6,000 rpm for 8 min. The cells collected were lyophilized with a vacuum freeze dryer (JOYN FD-1C-80, Shanghai Qiaoyue Electronics Co., Ltd), and the dry weight was measured. The method of Folch et al. (12) was used to determine the raw lipid content, and detailed procedures followed the method described by Lu et al. (13). A Vario EL Cube elemental analyzer (Elementar) was used to measure the percentage of C, H, N, S, and O in dry biomass. The formula outlined by Lesteur et al. (14) was used to evaluate the potential for producing biogenic methane (BMP_Th_), and the higher calorific values (HHVs) were calculated according to the method developed by Demirbaş (15). A 0.22 µm membrane was used to filter 10 mL of the sample solution, and an inductively coupled plasma mass spectrometer (Agilent Technologies 7700 series ICP/MS equipment) was used to determine the concentration of heavy metals.

For antibiotic determination, 80 mL of the sample solution was taken, frozen, and then dissolved in 5 mL of methanol. It was first pretreated using a C18 solid phase extraction column, then washed with methanol, dried almost completely, and finally dissolved in 1.5 mL of methanol. Before using, it was passed through a 0.22 µm filter membrane. The screening and quantitative determination of antibiotics (including 19 sulfonamides, 13 quinolones, and 4 tetracyclines) referred to the Chinese standard GB31658.17-2021, whereas the screening and qualitative determination of nine lincosamide antibiotics were guided by the standard GB/T 20762-2006. A system that included a liquid chromatography (Agilent 1290), a tandem mass spectrometer (Agilent 6495), and an AcclaimTM RSLC 120 C18 chromatographic column (2.2 µm, 2.1 × 100 mm) was utilized to identify antibiotics.

Initial BS CK0 (the same as CK on day 0), as well as control groups CK, HH01, and HS02, were sampled for metagenomic analysis on day 8. A 0.22 µm filter membrane was used to filter the samples. The filter membrane was rolled into a centrifuge tube following filtration. A DNeasy PowerWater Kit from the Mo Bio/QIAGEN company was then used to extract the samples. To determine the concentration of DNA, the absorbance values of DNA at 260 and 280 nm were measured using a Quant-iT PicoGreen dsDNA Assay Kit (P7589, Invitrogen) with a Quantifluoro-ST fluorometer (E6090, Promega). The required genomic library was created by following the guidelines for Illumina TruSeq DNA Sample Preparation.

To get quality-filtered reads for additional analysis, raw sequencing reads were processed. Cutadapt (version 1.2.1) was used to remove sequencing adapters from sequencing reads, and a sliding-window method in fastp was used to reduce low-quality reads. Following the acquisition of quality-filtered reads, metagenomics sequencing reads from each sample were taxonomically classified using Kaiju with greedy-5 mode against a nr-derived database and Kraken2 against a RefSeq-derived database. Each sample was assembled using Megahit (version 1.1.2) using the meta-large preset parameters. Contigs longer than 300 bp were then combined and clustered using mmseqs2 in “easy-linclust” mode, with the covered residues of the shorter contig set to 90%, and the sequence identity criterion set to 0.95. Contigs attributed to Viridiplantae or Metazoa were eliminated in the subsequent study after the non-redundant contigs were aligned against the NCBI-nt database using mmseqs2 in “taxonomy” mode to determine their lowest common ancestor taxonomy. The genes in the contigs were predicted using MetaGeneMark. CDS sequences of all the samples were clustered using mmseqs2’s “easy-cluster” mode, with the covered residue of the shorter contig set to 90%, and the protein sequence identity criterion set to 0.90. The high-quality reads from each sample were mapped onto the predicted gene sequences using salmon in the quasi-mapping-based mode with “--meta --minScoreFraction=0.55” to evaluate the abundances of these genes. The abundance values in metagenomes were then normalized using the copy per kilobase per million mapped reads.

The function of non-redundant genes was determined by annotating them using mmseqs2 in the “search” mode against the KEGG, EggNOG, and CAZy protein databases, respectively. EggNOG-mapper (version 2) was used to obtain EggNOG and GO. Map2slim was used to retrieve the GO ontology (www.metacpan.org). KOBAS was used to achieve KO. Using the default parameters, linear discriminant analysis effect size was used to identify differentially abundant taxa and functions between groups based on the taxonomic and functional profiles of non-redundant genes. Using principal coordinate analysis, nonmetric multidimensional scaling, and the unweighted pair-group method with arithmetic means hierarchical clustering, beta diversity analysis was carried out to examine the compositional and functional variation of microbial communities across samples using Bray-Curtis distance metrics.

### Statistical analysis

The experimental data were calculated and processed using Excel 2021, and statistical analysis was performed using SPSS 22. The mapping was completed using Excel 2021 and Origin Pro 2021 software.

## RESULTS AND DISCUSSIONS

### Microalgal growth in biogas slurry

**Fig 1:**
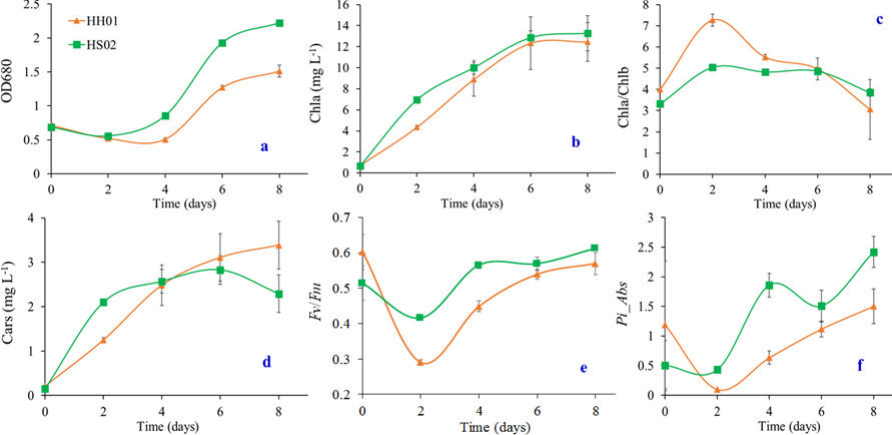
Changes in OD_680_ (**a**), Chl *a* (**b**), Chl *a*/Chl *b* (**c**), Cars (**d**), *F_v_/F_m_* (**e**), and *Pi-Abs* (**f**) during algal treatment of BS.

The values of OD_680_ (panel a), Chl *a* (panel b), Chl *a*/Chl *b* (panel c), Cars (panel d), *F_v_/F_m_* (panel e), and the performance index on absorption basis (*Pi_Abs*) (panel f) alterations when two algae strains handled BS are depicted in Fig. 1. The OD_680_ values for the algal treatments exhibit a trend of first declining and then rising, as seen in Fig. 1a. After day 4, the OD_680_ value of the HS02 treatment was noticeably higher than on the first day. However, the HH01 treatment’s hysteresis period persisted until day 4 (OD_680_ value was lower than on the first day). Figure S1 displays the algae growth status on day 8 of the experiment.

From day 0 to day 6, the Chl *a* concentrations of the HH01 and HS02 treatments exhibit a similar pattern of sharp rise (Fig. 1b). Between day 8 and day 6, there was no significant change in the concentration of Chl *a*. At every other sampling point, there was no significant difference in the Chl *a* concentrations of the two treatments, except for day 2, when the Chl *a* concentration of the HS02 treatment was higher than that of HH01. The specific growth rates for HH01 and HS02 treatments calculated based on Chl *a* concentration were 0.633 ± 0.056 day^−1^ and 0.680 ± 0.036 day^−1^, respectively, with no significant difference between the two treatments.

The Chl *a*/Chl *b* alterations are depicted in Fig. 1c. Prior to day 2, both HH01 and HS02 treatments showed an increase, and prior to day 4, the ratio for HH01 treatment was substantially greater than that of HS02 treatment. After hovering around 5 for the first 6 days, the ratio for HS02 treatment fell to about 4 on day 8. After the second day, the value for HH01 treatment steadily dropped until day 8. On days 6 and 8, there was no significant difference (*P* > 0.05) between the two treatments.

As shown in Fig. 1d, the Cars concentration of HS02 treatment rapidly increased before day 2, which was significantly higher than that of HH01 treatment, then gradually increased until day 6, after which it decreased and finally reached a concentration lower than HH01 on day 8. Remarkably, the Chl *a*, Chl *b*, and Cars concentrations of HS02 treatment were considerably greater than those of HH01 treatment on the second day. Furthermore, the pigment concentrations in the control group, which did not receive any algae treatment, were extremely low throughout the experiment; as a result, they were not depicted in the image.

The variations in *Fv/Fm* and *Pi_Abs* for HH01 and HS02 treatments are displayed in Fig. 1e and f, respectively. On the second day, both treatments’ *F_v_/F_m_* levels dropped, reaching 0.290 and 0.416, respectively, and the HH01 treatment’s decline was larger. The value then progressively rose under both treatments. At every other sampling point, the *F_v_/F_m_* values of the HS02 treatment were greater than those of the HH01, except for days 1 and 8. The *F_v_/F_m_* values of the BS treated with HH01 and HS02 were 0.569 and 0.612 on day 8, respectively. Nevertheless, there was no significant difference between the two therapies.

Except for the fact that on day 8, the *Pi_Abs* value in the HS02 treatment was noticeably greater than that in the HH01 treatment, the changing trend of *Pi_Abs* in the two treatments was in line with *F_v_/F_m_*. The pigment concentration and chlorophyll fluorescence parameter values above suggest that HS02 is more capable of adapting to BS than HH01.

Studies have shown that some microorganisms or high concentrations of NH_4_^+^-N or high turbidity in pig farm BS may significantly inhibit the growth of microalgae (16). BS normally needs to be diluted during the cultivation of microalgae. For example, Tang et al. (17) discovered that *Didymogenes chengda* CDU-W13 cannot grow at NH_4_^+^-N concentrations higher than 300 mg L^−1^. The two strains of algae isolated from the activated sludge of a sewage plant in this study can grow in unpretreated and undiluted pig farm digestate, indicating extremely high tolerance of the microalgae to BS. The trend of microalgal OD value, *F_v_/F_m_*, and *Pi_Abs* decreasing first and then increasing in 0–4 days indicates the adaptation process of microalgae to the new environment when they first enter the digestate system.

Chl *a*, the primary chlorophyll found in single-cell microalgae, is correlated with photosynthetic rate and chloroplast activity. Chl *a*’s high increase suggests that microalgae can develop in BS while coexisting with native microbes. Following the second day, the HS02 treatment group’s *F_v_/F_m_* and *Pi_Abs* values were higher than those of HH01, suggesting that BS had a stronger inhibitory effect on photosystem II activity in the HH01 group than in the HS02 group.

### The ability of microalgae to remove pollutants from biogas slurry

**Fig 2:**
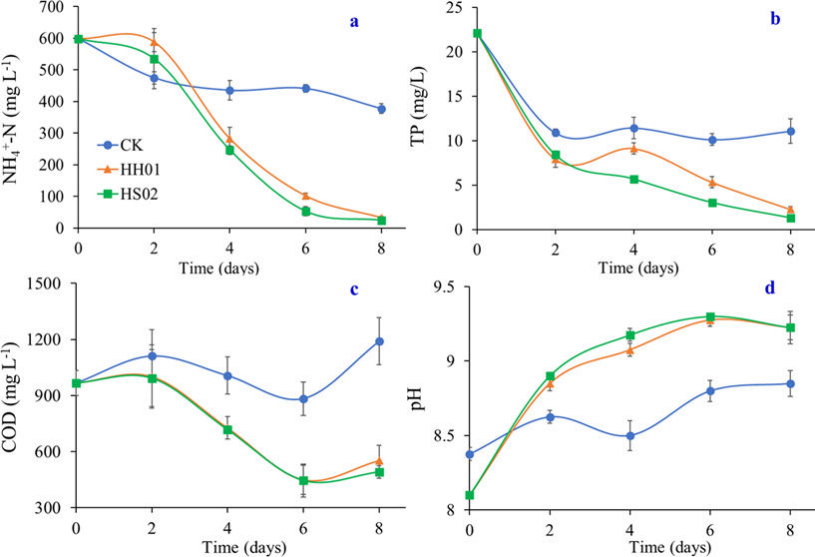
Changes in NH_4_^+^-N (**a**), TP (**b**), COD (**c**) concentrations, and pH (**d**) of BS under different treatments.

The variations in NH_4_^+^-N concentration under various treatments are depicted in Fig. 2a. The initial NH_4_^+^-N concentration of the BS was very high (598 mg L^−1^), and in the CK group, this concentration gradually dropped. From day 0 to day 2, the NH_4_^+^-N concentration in the HH01 and HS02 treatments declined gradually. However, after day 2, both treatments saw a sharp decline. With removal rates of 94.6% and 95.9%, respectively, the NH_4_^+^-N concentrations in the H01 and HS02 treatments were only 32.6 and 24.3 mg L^−1^ on day 8, and there was no significant difference between the two treatments. In the current study, the NH_4_^+^-N elimination rate is higher than the average level found in the literature, where the baseline NH_4_^+^-N level in BS was lower (2, 7, 18–20). As illustrated in Fig. 2b, the BS had an initial TP concentration of 22.1 mg L^−1^, and the TP concentration in the CK group varied between 10.1 and 11.4 mg L^−1^ from 2 to 8 days after rapidly declining from 0 to 2 days. It first rose and then fell in the HH01 treatment, whereas it kept falling in the HH02 treatment. Following 8 days of treatment, the removal rates were 89.7% and 94.0%, respectively, and the TP concentrations were 2.27 and 1.32 mg L^−1^ for HH0 and HS02 treatments, respectively. It has been demonstrated that two microalgal strains exhibit a moderate to high rate of TP removal from BS (2, 7, 18–20). In a different study, Zhang et al. (10) treated unsterilized pig effluent with *Chlorella pyrenoidosa*, achieving removal rates of 98.7% for NH_4_^+^-N and 42.8% for TP. Nevertheless, NH_4_^+^-N and TP concentrations were only 11.6 and 7.8 mg L^−1^, respectively.

Microalgae offer special contributions to the removal of N and P from BS. Through absorption and assimilation, microalgae may efficiently extract N from wastewater (21). Microalgae preferentially use NH_4_^+^-N over NO_3_-N and organic N for the direct synthesis of compounds like proteins and amino acids (22). Furthermore, the activity of other microorganisms may cause biological nitrification in the non-sterile BS, and the two processes together may accelerate the drop in NH_4_^+^-N concentration in BS (1, 18). However, it should be mentioned that in high irradiance circumstances, microalgal proliferation can hinder denitrification (23). Algal cells can directly absorb phosphates, which can then be transformed by phosphorylation pathways into ATP and phospholipids (1). In addition, it should be mentioned that the growth of algae may significantly aid in the precipitation removal of P due to the rise in pH of the culture medium (Fig. 2d).

With an initial COD value of 967 mg L^−1^, Fig. 2c illustrates the variations in the BS’s COD concentration. From day 0 to day two, there is a modest increase in the COD content in the CK group, which then steadily declined until day 6 before rising once more on day 8. The COD changes for the two algal strain treatments were nearly the same, showing virtually little change from days 0 to 2 before rapidly declining to their lowest values on day 6 and then slightly increasing on day 8. On day 8, there was no significant difference between the COD clearance rates for the HH01 and HS02 treatments, which were 42.9% and 49.2%, respectively.

Through photosynthesis, microalgae primarily take up carbon dioxide (CO_2_) from the atmosphere. The microalgae enzyme ketose diphosphate carboxylase fixes CO_2_, and the Calvin cycle transforms it into organic materials (1, 24). Additionally, certain microalgae can obtain carbon from organic substances (1, 23). However, our preliminary investigation revealed that HH01 and HS02 had little to no growth in glucose-containing medium (data not shown), suggesting that their capacity to remove COD was either nonexistent or very limited. The release of organic photosynthate into BS by microalgae during the cultivation stage could be the cause of the rise in COD at 6–8 days (7, 18). It is hypothesized that the key factors in the removal of COD may be the oxygen and particular growth-promoting compounds that algae release to boost bacterial activity. The COD removal rate of HS02 treatment was slightly higher than that of *Parachlorella kessleri* QWY28 (47%) and *Desmodesmus* sp. QWY36 (48%), as reported by Qu et al. (16).

As can be seen in Fig. 2d, the pH in the CK groups gradually rose over time after declining somewhat on day 4. As the duration increased, the pH of the algal treatments rose quickly, peaking on day 6 but declining somewhat on day 8. Nevertheless, the end pH of both groups was 9.2, which was noticeably higher than the CK group (8.9). Bicarbonate ions were pumped into the cell to supply CO_2_ for photosynthesis, whereas hydroxide ions were pumped out of the cell, which is why the pH value increased with algal treatment (25).

While microalgae may remove heavy metals from BS through surface precipitation, absorption, covalent bonding, ion exchange, and adsorption, they can also eliminate antibiotics by sorption, accumulation, and degradation (1, 26, 27). Table 1 shows the variations of metal and antibiotic concentrations in BS under different treatment circumstances. According to Table 1, HH01 treatment demonstrated the best removal impact on Ag from BS, with a removal rate of up to 93.4%, followed by Cd (66.1%), Mn (58.4%), and Fe (57.4%), but exhibited lesser removal ability for Ni. Ag was most effectively removed by HS02 treatment (92.8%), followed by Mn (74.7%) and Fe (67.9%), whereas Cu and Ni were less efficiently removed. Despite low As concentrations, neither algal strain exhibited detectable removal from BS. Algal cells can absorb metals like Mn, Cu, Co, Ni, Zn, Cr, and Fe, which are essential for controlling the fundamental cellular processes of many different organisms (28). According to Leong and Chang (29), even in trace concentrations, As, Cd, Cr, Pb, and Hg are hazardous and carcinogenic, endangering both human health and the ecosystem. In wastewater containing 1.85 mg L^−1^ of Zn (II) and 1 or 6 mg L^−1^ of Mn (II), Liu et al. (30) discovered that *Chlorella vulgaris* had the highest removal rates for Zn (II) (86.72% and 97.16%, respectively) and Mn (II) (42.74% and 30.33%, respectively). It should be noted that the increase in pH of the culture medium caused by the growth of algae may greatly facilitate the removal of heavy metals by precipitation. As seen in Fig. 2d, the pH value of the algae treatment increased rapidly, ultimately reaching 9.2.

**TABLE 1:** Concentrations of metal and antibiotic in different treatments of biogas slurry (μg L^−1^)^*a*^

Metal or antibiotic	Treatment		
	CK	HH01	HS02
Fe	153.8 ± 19.1a	65.6 ± 26.6b	49.5 ± 5.8b
Zn	84.7 ± 2.8a	56.4 ± 11.3b	40.0 ± 9.6b
Cu	74.4 ± 0.3a	57.4 ± 4.1b	67.9 ± 1.2b
Mn	41.1 ± 0.2a	17.1 ± 8.8b	10.4 ± 8.6b
Ni	29.3 ± 0.2a	24.6 ± 2.2b	25.2 ± 0.9b
Cr	6.2 ± 0.1a	4.0 ± 0.2b	4.1 ± 0.0b
As	2.17 ± 0.03a	2.23 ± 0.07a	2.18 ± 0.06a
Pb	1.25 ± 0.74a	1.62 ± 1.08a	1.26 ± 1.04a
Ag	0.72 ± 0.24a	0.05 ± 0.02b	0.05 ± 0.01b
Cd	0.10 ± 0.01a	0.06 ± 0.03b	0.05 ± 0.01b
Sulfisomidine	1.30 ± 0.11a	0.96 ± 0.08b	0.91 ± 0.07b
Sulfamethazine	1.39 ± 0.12a	1.05 ± 0.15b	0.97 ± 0.09b
Lincomycin	1.42 ± 0.02a	0.87 ± 0.05b	1.00 ± 0.08b

^*a*^ Different letters in the same row indicate significant differences between treatments (*P* < 0.05).

Of the antibiotics that were screened, only lincomycin, sulfisomidine, and sulfamethazine were found in the BS. The removal rates for lincomycin, sulfisomidine, and sulfamethazine under HH01 treatment were 38.6%, 26.0%, and 18.5%, respectively. All three of these antibiotics had removal rates of about 30% under HS02 treatment. According to Peng et al. (31), *C. vulgaris* eliminated 32.06%, 31.17%, and 34.07% of sulfadiazine, sulfamethazine, and sulfamethoxazole, respectively, from aquaculture effluent after 12 days of cultivation. This removal ability is comparable to that of HS02 in this study (30%). Xiong et al. (32) used *Scenedesmus obliquus* grown for 11 days to remove 17.3% of sulfamethazine and 29.3% of sulfamethoxazole.

While COD levels are close to the national livestock and poultry breeding industry pollutant discharge standard (GB18596-2001) (400 mg L^−1^), they are significantly higher than the latest Guangdong livestock and poultry breeding industry pollutant discharge standard (DB44/613-2024) (100–150 mg L^−1^). However, the concentrations of NH_4_^+^-N, TP, and heavy metals all meet the standards set by GB18596-2001 and DB44/613-2024, indicating the treatment effect of BS. The future tendency is to further lower the COD limit. Therefore, this technology must be used in conjunction with other technologies to improve COD removal.

### Assessment of the bioenergy production potential of biomass

Table 2 displays the algal biomass, lipid production, elemental composition, and methane production potential (BMP_Th_) under various treatments. The productivity of HH01 and HS02 treatments was 65.5 mg total suspended solids (TSS) L^−1^ day^−1^ and 73.9 mg TSS L^−1^ day^−1^, respectively, after 8 days of treatment. This was significantly less than the productivity of 788 mg L^−1^ day^−1^ reported by Qu et al. (16), who carried out a similar study with undiluted and non-sterile pig farm BS. With lipid content of 8.5% and 10.4% and lipid productivity of 5.6 and 7.7 mg L^−1^ day^−1^, respectively, the HH01 and HS02 treatments differed significantly from one another. Compared to HH01, HS02 treatment demonstrated a noticeably greater capability for lipid synthesis. Compared to HH01 treatment, the C, O, N, C/N ratio, and BMP_Th_ values for HS02 treatment were substantially greater. The HHV for HH01 and HS02 treatments was 16.5 ± 0.4 and 14.3 ± 2.8 MJ kg^−1^, respectively, while the BMP_Th_ values were 179 and 204 mL g^−1^ VS, respectively (Table 2).

**TABLE 2:** Algal biomass, lipid production, elemental composition, methane production potential (BMP_Th_), and HHV under different treatments

	Biomass yield (mg L^−1^)	Biomass productivity (mg L^−1^ day^−1^)	Lipid content (%)	Lipid productivity (mg L^−1^ day^−1^)	N (%)	C (%)	H (%)	S (%)	O (%)	C/N	BMP_Th_ (mL g^−1^ VS)	HHV (MJ kg^−1^)
HH01	524 ± 54	65.5 ± 6.8	8.5 ± 0.6	5.6 ± 0.8	6.6 ± 0.1	34.1 ± 0.5	6.2 ± 0.2	0.5 ± 0.0	29.9 ± 0.4	5.2 ± 0.0	179 ± 2	16.5 ± 0.4
HS02	591 ± 60	73.9 ± 7.5	10.4 ± 0.1^*a*^	7.7 ± 0.8^*a*^	6.8 ± 0.1^*a*^	36.2 ± 0.0^*a*^	6.4 ± 0.2	0.5 ± 0.0	28.9 ± 0.2^*a*^	5.3 ± 0.1^*a*^	204 ± 2^*a*^	14.3 ± 2.8

^*a*^ indicates significant differences between treatments (*P* < 0.05).

Both lipid content and lipid productivity are significantly lower than those reported in the literature (1), indicating that the biomass collected cannot be used for biodiesel production. The HHV and BMP_Th_ values for the HH01 and HS02 treatments are close to those reported in the literature (7, 33), indicating that the biomass still has certain application value in the production of bioenergy.

### Microbial metagenomic analysis of biogas slurry

**Fig 3:**
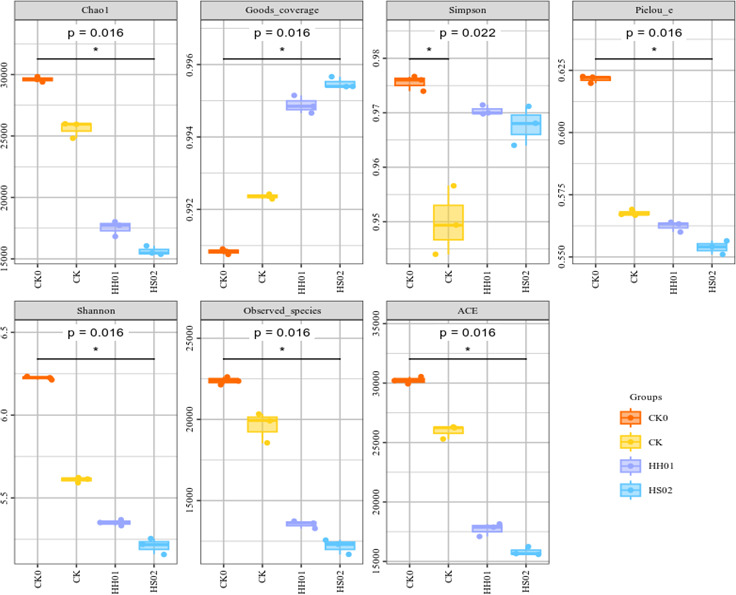
Alpha diversity index analysis of species.

Figure 3 displays the findings of the Alpha diversity index analysis of species for microorganisms in BS under various treatments. The ACE index, observed species, Chao1, Shannon, and Pielou-e all display the same pattern in the figure, namely that the size order of the four treatments is CK0 > CK > HH01 > HS02, whereas Goods_coverage displays the reverse trend. While not substantially different from HH01 treatment, the Simpson index was significantly higher in CK treatment compared to HS02 and CK0. In comparison to CK0 (original BS), 8 days of light treatment (CK) decreased the microbial variety, and the addition of microalgae further decreased the microbial diversity. Following algal treatment, there was a declining trend in the number of species, Chao1 index, ACE index, and Shannon index, suggesting that algal treatment decreased the microbial species in the BS, which in turn led to a decline in microbial richness and diversity. This is in line with the findings of Sun et al. (34), who demonstrated that the bacteria in the algae-sludge membrane bioreactor exhibited the lowest microbial richness and diversity (the Simpson index rose, and the Shannon index fell). Qu et al. (35) also discovered that raw swine wastewater treated with *Chlorella* sp. MA1 and *Coelastrella* sp. KE4 shows a considerable increase in the Simpson index and a sharp decline in the Chao1 and Shannon indices. By inoculating microalgae, the relationship between bacteria and algae may be changed, resulting in an increase in some low-abundance bacteria and the suppression or even extinction of some species (34–36). However, Bankston et al. (18) found that algal treatment resulted in a slightly higher Shannon diversity and evenness in BS containing activated sludge, while the *C. sorokiniana* treatment had no significant effect on these parameters in the native poultry litter BS community when treated with anaerobic digestate.

**Fig 4:**
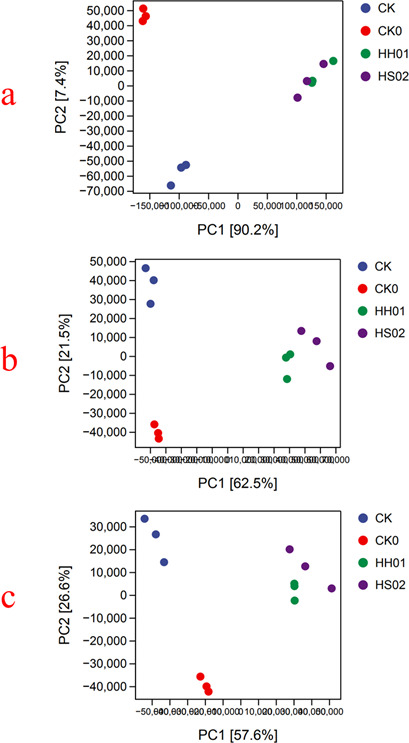
PCA results at the phylum (**a**), genus (**b**), and species (**c**) levels.

The PCA at the phylum level (panel a), genus level (panel b), and species level (panel c) is displayed in Fig. 4. According to Fig. 4, CK and CK0 are grouped in distinct, widely separated quadrants, but HH01 and HS02 treatments are quite close to one another and essentially in the same quadrant, widely separated from CK and CK0. This implies that although there is no significant difference between the two algal treatments, the community structure of the algal-treated BS differs significantly from that of CK and CK0.

The Venn diagram of variations in microbial composition is displayed in Fig. S2. The original total number of microbial phyla in the BS for CK0 was 211, as shown in Fig. S2a. Eight days later, there were 213, 190, and 176 phyla in BS for CK, HH01 treatment, and HS02 treatment, respectively. The CK group, HH01 treatment, and HS02 treatment had 205, 186, and 173 identical phyla compared to CK0, respectively. The original total number of microbial genera in the BS for CK0 was 12,891, as shown in Fig. S2b. Eight days later, there were 12,080, 8,552, and 7,647 genera in the BS for CK, HH01 treatment, and HS02 treatment, respectively. The CK group, HH01 treatment, and HS02 treatment had 11,456, 8,283, and 7,439 identical genera compared to CK0, respectively. In the initial BS (CK0), 32,035 species were found, as illustrated in Fig. S2c. After 8 days, there were 28,193 species in the CK group, which was not significantly different from CK0. Algal treatment dramatically decreased the number of species in the BS, as seen by the 17,901 and 15,821 species found in the BS of the HH01 and HS02 treatments, respectively. Only 14,891 species were the same between HS02 and CK0 after 8 days, compared to 25,898 between CK and CK0 and 16,674 between HH01 treatment and CK0. In conclusion, HS02 > HH01 > CK is the trend of microbial composition variations throughout treatments.

Proteobacteria, Cyanobacteria, Bacteroidetes, and Firmicutes typically make up the microbial composition of pig farm wastewater, with Patescibacteria, Actinobacteria, Chloroflexi, Planctomycetes, and Euryarchaeota following (1). Among them, some members of the Bacteroidetes and Proteobacteria can remove organic chemicals and NH_4_^+^-N (37), while some members of the Firmicutes are heterotrophic denitrifiers and degraders of volatile fatty acids (38, 39).

**Fig 5:**
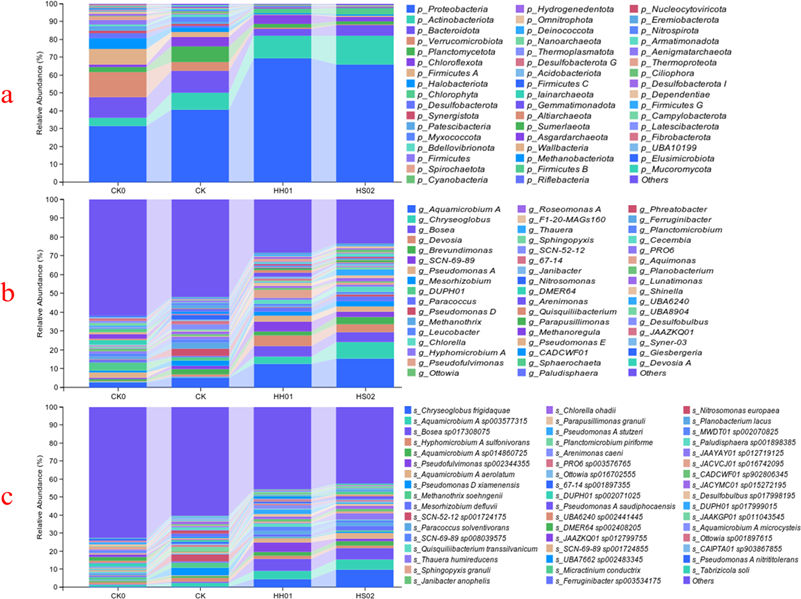
Stacked bar charts of species composition at different levels: phylum (**a**), genus (**b**), and species (**c**) (top 50). CK0 and CK represent the species composition of BS treated for 0 and 8 days, respectively, while HH01 and HS02 represent the species composition of algae HH01 and HS02 treated for 8 days, respectively.

The stacked bar charts of the microbial composition for the various treatments are displayed in Fig. 5. As shown in Fig. 5a, the top 50 phyla for each treatment and the top six dominant phyla for different treatments are CK0 (Proteobacteria, 30.8%; Verrucomicrobiota, 12.9%; Bacteroidota, 11.0%; Firmicutes A, 10.0%; Actinobacteriota, 5.3%; and Halobacteriota, 4.9%), CK (Proteobacteria, 40.9%; Bacteroidota, 11.2%; Planctomycetota, 10.2%; Actinobacteriota, 10.0%; Verrucomicrobiota, 5.1%; and Chloroflexota, 4.2%), HH01 (Proteobacteria, 70.5%; Actinobacteriota, 12.4%; Bacteroidota, 3.6%; Chloroflexota, 3.3%; Planctomycetota, 2.8%; and Chlorophyta, 2.5%), and HS02 (Proteobacteria, 60.1%; Actinobacteriota, 14.2%; Bacteroidota, 5.7%; Chlorophyta, 3.5%; Planctomycetota, 2.1%; and Chloroflexota, 1.8%). The most prevalent phylum across all treatments was Proteobacteria, and following algal treatments, its abundance dramatically rose. Actinobacteriota rose to the second-most abundant phylum at the same time, and naturally, Chlorophyta abundance also rose. Nonetheless, there was a notable decline in the abundance of Verrucomicrobiota and Bacteroidota.

The top 50 genera for each treatment are displayed in Fig. 5b, with *Aquamicrobium* being the most prevalent across all treatments. *Aquamicrobium*, *Pseudomonas*, *Leucobacter*, *Ottowia*, *Methanothrix*, *Mesorhizobium*, *Brevundimonas*, *Paracoccus*, *Hyphomicrobium*, and *Sphingopyxis* were the top 10 genera found in the original BS. This was not consistent with findings from other investigations that found the most common genera in swine wastewater were *Blastomonas*, *Flavobacterium*, *Skermanella*, *Macromonas*, *Calothrix*, and *Sedimentibacter* (1). *Aquamicrobium*, *Devosia*, *Bosea*, *Chryseoglobus*, *Chlorella*, *Pseudomonas*, *Mesorhizobium*, *Brevundimonas*, and *Paracoccus* were among the top nine genera found in the BS following HH01 or HS02 treatment.

The relative abundance of genera such as *Aquamicrobium*, *Devosia*, *Bosea*, *Chryseoglobus*, and *Chlorella* increased because of algal treatments, but *Leucobacter*, *Ottowia*, and *Methanothrix* saw a large decline. The majority of *Aquamicrobium* species have been isolated from habitats that are polluted, and they can function as bacteria that degrade phenolic substances or oxidize sulfur (40, 41).

The top 50 species for each treatment are displayed in Fig. 5c. *Pseudomonas xiamenensis*, *Methanothrix soehngenii*, *Aquamicrobium* sp. 003577315, *Hyphomicrobium sulfonivorans*, *Aquamicrobium* sp. 014860725, *Sphingopyxis granuli*, *Mesorhizobium defluvii*, *Janibacter anophelis*, *Bosea* sp., and *Aquamicrobium aerolatum* were the top 10 species found in the original BS. Following algal treatments, the abundance of some species, such as *Pseudofulvimonas* sp., *Bosea* sp., *Chryseoglobus frigidaquae*, *A. aerolatum*, *Thauera humireducens*, and *Chlorella ohadii*, increased noticeably. The bacterium *Methanothrix soehnenii*, which produces methane, was the species that declined the most. It implies that methane-producing bacteria can be inhibited by algal treatment.

According to the current study, microalgae appear to function as activators to increase the activity of beneficial microbes that treat BS. This is in line with the findings of Bankston et al. (18), who discovered that when treating poultry litter BS, *Chlorella sorokiniana* supports the populations of nitrifying, phosphate-accumulating, and heterotrophic bacteria.

The relative abundance of the top 20 pathogens in BS is displayed in Table 3. *Salmonella enterica*, *Pseudomonas aeruginosa*, *Staphylococcus aureus*, *Escherichia coli*, *Acinetobacter baumannii*, *Mycobacterium tuberculosis*, and *Streptococcus pneumoniae* were shown to be the most prevalent pathogens in BS before treatment. All pathogen abundance decreased because of microalgal treatment. The mean decrease value of HS02 treatment was greater than that of HH01 treatment, but the difference between the two treatments was not statistically significant. The pathogens *S. enterica*, *P. aeruginosa*, *S. aureus*, *E. coli*, *A. baumannii*, *M. tuberculosis*, and *S. pneumoniae* showed 78.3%, 76.1%, 81.4%, 77.1%, 78.6%, 72.8%, and 78.0% decreases in abundance for HS02 treatment, respectively. Many pathogens were far less abundant in CK than in CK0, but both were much more abundant than in the algal treatment. Eliminating pathogens from wastewater is a crucial objective in wastewater treatment to lower health hazards and stop the spread of disease (42).

**TABLE 3:** Changes in the relative abundance of the top 20 pathogenic microorganisms in BS^*a*^

	CK0	CK	HH01	HS02
*Salmonella enterica*	10.25 ± 0.44a	7.55 ± 1.83b	2.84 ± 0.59c	2.22 ± 0.30c
*Pseudomonas aeruginosa*	9.86 ± 0.42a	7.53 ± 2.05b	3.08 ± 0.78c	2.36 ± 0.35c
*Staphylococcus aureus*	8.44 ± 0.32a	5.83 ± 1.39b	1.93 ± 0.41c	1.57 ± 0.21c
*Escherichia coli*	7.92 ± 0.35a	5.83 ± 1.50b	2.30 ± 0.53c	1.81 ± 0.28c
*Acinetobacter baumannii*	4.01 ± 0.12a	2.90 ± 0.70b	1.03 ± 0.23c	0.86 ± 0.10c
*Mycobacterium tuberculosis*	3.89 ± 0.17a	3.32 ± 1.03a	1.25 ± 0.23b	1.06 ± 0.18b
*Streptococcus pneumoniae*	3.77 ± 0.16a	2.59 ± 0.47b	1.08 ± 0.22c	0.83 ± 0.09c
*Klebsiella pneumoniae*	0.37 ± 0.02a	0.30 ± 0.07b	0.11 ± 0.02c	0.09 ± 0.01c
*Aspergillus fumigatus*	0.33 ± 0.01a	0.24 ± 0.06b	0.09 ± 0.02c	0.07 ± 0.01c
*Erwinia amylovora*	0.32 ± 0.02a	0.25 ± 0.06a	0.10 ± 0.02b	0.08 ± 0.01b
*Streptococcus suis*	0.31 ± 0.01a	0.24 ± 0.10a	0.07 ± 0.01b	0.05 ± 0.01b
*Listeria monocytogenes*	0.28 ± 0.01a	0.19 ± 0.05b	0.06 ± 0.01c	0.05 ± 0.01c
*Vibrio cholerae*	0.26 ± 0.01a	0.18 ± 0.04b	0.07 ± 0.02c	0.06 ± 0.01c
*Pseudomonas syringae*	0.23 ± 0.01a	0.18 ± 0.04b	0.08 ± 0.03c	0.06 ± 0.01c
*Cryptococcus neoformans*	0.24 ± 0.01a	0.18 ± 0.04b	0.06 ± 0.02c	0.05 ± 0.01c

^*a*^ Different letters in the same row indicate significant differences between treatments (*P* < 0.05).

*Clostridium* sp., *Escherichia* sp., *Chryseobacterium* sp., *Pseudomonas* sp., *Salmonella* sp., *Advenella* sp., and *Arcobacter* sp. are often the most common pathogenic bacteria found in actual swine wastewater, and their numbers dramatically dropped following microalgal treatment (1, 35).

For instance, in the final effluent of the combined purification system with *Scenedesmus* sp. for pig farm BS treatment, Caballero-Lajarín et al. (43) noted notable decreases for *Salmonella*, *Shigella*, and *E. coli*. Xinjie et al. (44) found that co-cultivation of vetiver and green alga *Dictyphosphaerium* sp. can inactivate pathogens in pig wastewater, and the highest removal rates for *Escherichia* spp., *Arcobacter* spp., *Clostridium* spp., *Chryseobacterium* spp., and *Pseudomonas* spp. were 100%, 60%, 99.96%, 52%, and 90%, respectively. *Advenella* sp., *Arcobacter* sp., *Bacillus* sp., and *Staphylococcus* sp. were found to be prevalent in raw swine wastewater by Qu et al. (35). Following an 8-day treatment period, the operational taxonomic units for most pathogens experienced a significant decrease, with 29 and 22 of the pathogenic genera showing no detectable presence in the presence of *Chlorella* sp. MA1 and *Coelastrella* sp. KE4, respectively. Similar to the findings of Caballero-Lajarín et al. (43), our research shows that the pathogen with the highest abundance is *S. enterica*. Following microalgal treatments, microbial antagonism, nutritional competition (N and P removal, pH promotion, O_2_ release, toxins generation, etc.), and phagocytosis may contribute to the decline in pathogen abundance (23, 36).

**Fig 6:**
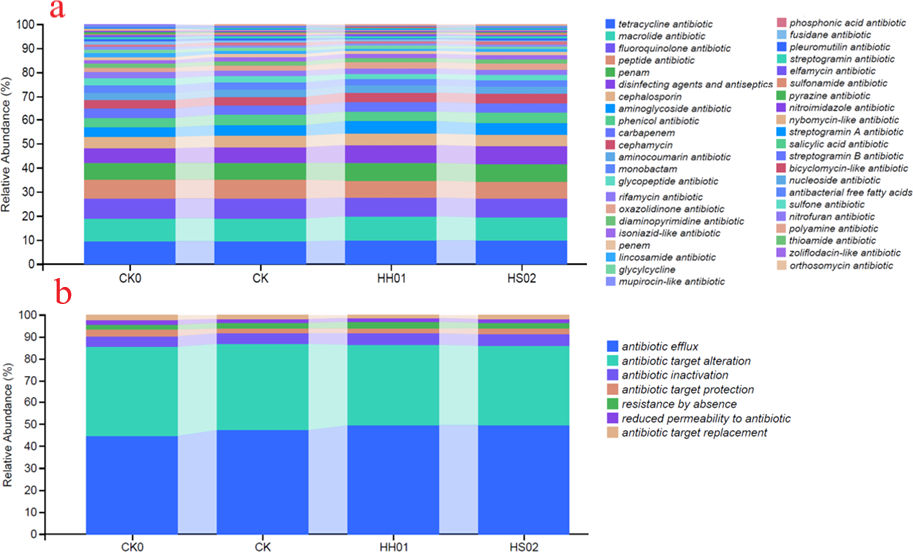
Alterations in drug class (**a**) and resistance mechanism (**b**) of antibiotic resistance genes across different treatment conditions.

Microorganisms in the BS samples carried up to 305 antibiotic resistance genes (ARGs), spanning 43 types of antibiotic resistance, including tetracycline, macrolide, fluoroquinolone, peptide, and penam, disinfecting agents, and antiseptics, as shown in Fig. 6a. Wang et al. (45) reported a high abundance of ARGs in pig farms and their surrounding soils and water, indicating that anaerobic digestion has limited effectiveness in reducing ARGs. Compared to the CK0 treatment, the HS02 treatment showed a significant reduction (>10%) in ARG abundance for fusidane (36.0%), mupirocin-like (35.2%), glycylcycline (27.9%), glycopeptide (26.2%), pleuromutilin (24.6%), rifamycin (14.8%), and peptide (12.1%) (percent reduction in parentheses). Conversely, ARG abundance significantly increased (>10%) for oxazolidinone (45.5%), disinfecting agents and antiseptics (21.1%), aminoglycoside (20.2%), isoniazid-like (17.4%), and diaminopyrimidine (17.2%) (percent increase in parentheses) (Table S2; Fig. 6a). Notably, tetracycline resistance genes, which had the highest relative abundance in CK0, increased significantly by 5.3%, while macrolide resistance genes, the second most abundant, showed no significant change. Additionally, lincosamide and sulfonamide resistance genes, associated with the detected sulfonamide and lincosamide antibiotics, exhibited relatively low abundance but were significantly reduced. This further demonstrates that the concentration of different antibiotics does not strictly correlate with the abundance of induced ARGs (45, 46). Few studies have reported microalgae-based treatments reducing ARGs in wastewater. For example, Cheng et al. (47) found that algal wastewater treatment with *Galdieria sulphuraria* significantly removed ARGs. Cheng et al. (48) showed that algae-based systems not only eliminated most ARGs in treated water compared to traditional activated sludge systems but also effectively narrowed the host range of ARGs. However, a dual effect (removal and enrichment) of algal treatment on ARGs was observed in BS. This study observed a significant increase in ARGs for certain antibiotic types after microalgae treatment (Table S2; Fig. 6a). This phenomenon may be attributed to aerobic bacteria carrying these ARGs. Anaerobic fermentation in pig manure suppresses such bacteria, while the aerobic environment in algae-enhanced treatments likely promotes their rapid proliferation, leading to increased ARG abundance (45, 49).

These ARGs encompassed seven resistance mechanisms, with antibiotic efflux (46.9%) and target alteration (39.3%) being predominant across samples (Fig. 6b). Wang et al. (45) identified antibiotic efflux as the primary resistance mechanism (60%–66%) in microbial communities hosting ARGs in swine farms and surrounding environments, linking it to *Pseudomonas* species encoding efflux pump genes. In this study, *Pseudomonas* ranked second in microbial community abundance (Fig. 5b), with *P. xiamenensis* exhibiting the highest species-level abundance (Fig. 5c).

**Fig 7:**
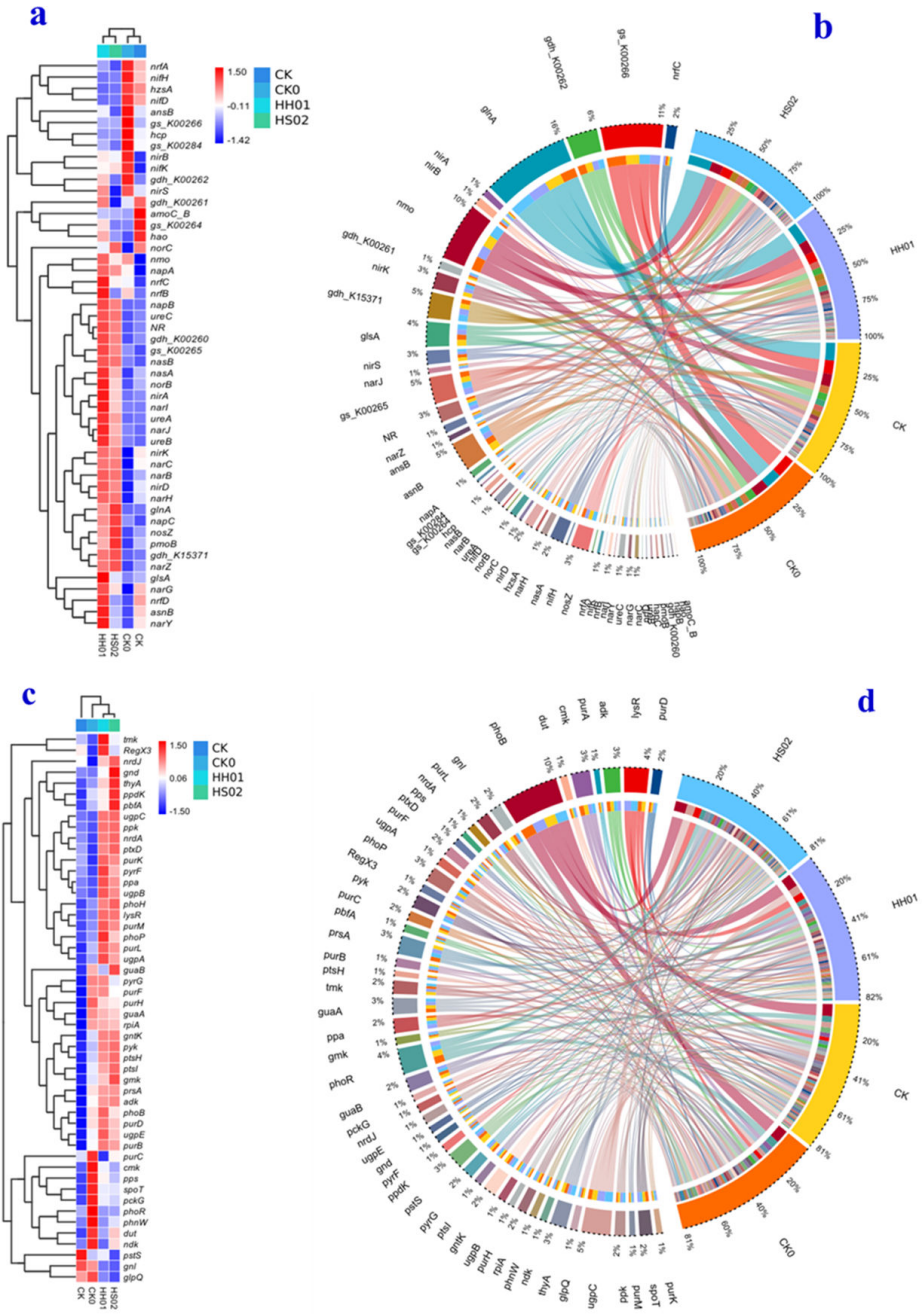
Differential heat map of N cycle-related gene expression (**a**), circos map of N cycle-related gene composition (**b**), differential heat map of P cycle-related gene expression (**c**), and circos map of P cycle-related gene composition (**d**) in different treatments of BS microorganisms.

Figure 7 shows the differential heat maps of N cycle (panel a) and P cycle (panel c) related gene expression and circular maps of N cycle (b) and P cycle (d) related gene composition of BS microorganisms in different treatments. As shown in Fig. 7a and b, more than 20 genes linked to N cycling, such as *napA*, *narl*, *nasA*, *narC*, *pmoB*, *narB*, *narH*, *nirK*, *nirD*, *narJ*, and *napC*, showed a significant increase in abundance when compared to CK. In contrast, the abundance of genes like *nirs*, *hzsA*, *nifD*, *nifH*, *nrfA*, *hcp*, *gs_K00284*, *gdh-K00262*, *gs_K00266*, and *ansB* significantly decreased. As shown in Fig. 7c and d, more than 20 P-cycling-related genes, such as *nrdJ*, *nrdA*, *pyrF*, *ptxD*, *purK*, *ugpC*, *ppk*, *ppa*, *ugpB*, *pbfA*, *gnd*, *gmk*, *thyA*, *ppdK*, *pyk*, and *adk*, showed a significant increase in abundance when compared to CK, whereas the abundance of several genes, including *pstS*, *gnl*, and *glpQ*, significantly decreased. This implied that HH01 and HS02 treatments improved N and P removal by increasing the number of genes linked to the N and P cycles in microorganisms.

### Conclusion

The concentrations of pollutants such as NH_4_^+^-N, TP, COD, and heavy metals in non-sterile BS were considerably decreased following treatment with the two microalgae strains HH01 and HS02. Metagenomic research indicates that the number of species, richness, variation, and pathogens within BS microorganisms were significantly reduced by algae treatment. Meanwhile, the relative abundance of genes involved in microbial cycling of N and P increased significantly. It seems that the microalgae operate as activators, increasing the activity of beneficial bacteria for BS treatment. However, it should be highlighted that the microorganisms in BS include a high number of ARGs. Although microalgal treatment reduced the abundance of some ARGs, others increased significantly. These findings provide advanced insights into how microalgae affect the microbial composition and ARGs within unsterilized pig farm BS.

## Data Availability

All metagenomic sequences of microbiota in biogas slurry under various treatments are available in the NCBI Sequence Read Archive under BioProject accession number PRJNA1302027.

## SUPPLEMENTAL MATERIAL

Supplemental material spectrum.00688-25-s0001.docx Tables S1 and S2; Fig. S1 and S2.

ASM does not own the copyrights to Supplemental Material that may be linked to, or accessed through, an article. The authors have granted ASM a non-exclusive, world-wide license to publish the Supplemental Material files. Please contact the corresponding author directly for reuse.
