# Supplementary material for: Treatment of non-sterile biogas slurry from a pig farm using microalgae isolated from the activated sludge of sewage plants
Source: Microbiol Spectr. 2025 Sep 9;13(10):e00688-25. doi: 10.1128/spectrum.00688-25 (PMC12502765; doi:10.1128/spectrum.00688-25)
Supplement: Supplemental material — Tables S1 and S2; Fig. S1 and S2. [file spectrum.00688-25-s0001.docx]

Table S1 Blast results of 18S rRNA of two microalgae

|  | Description | Max score | Total score | Query cover | E | Ident% | Accession |
| --- | --- | --- | --- | --- | --- | --- | --- |
| HH01 | *Chlorella sorokiniana* isolate Zhaodong Salt Lake 2 small subunit ribosomal RNA gene, partial sequence | 2944 | 2944 | 100% | 0 | 99.9 | MK764916.1 |
| HS02 | *Chlorella sorokiniana* 18S ribosomal RNA gene, partial sequence | 3013 | 3013 | 100% | 0 | 99.8 | KP771817.1 |

Table S2 The abundance of resistance genes of different classes of antibiotics in microbial communities of BS under different treatments

| Term | CK0 | CK0 | CK0 | CK | CK | CK | HH01 | HH01 | HH01 | HS02 | HS02 | HS02 |
| --- | --- | --- | --- | --- | --- | --- | --- | --- | --- | --- | --- | --- |
| tetracycline antibiotic | 0.0961 | 0.0958 | 0.0949 | 0.0953 | 0.0979 | 0.0956 | 0.1010 | 0.1038 | 0.0989 | 0.0989 | 0.1015 | 0.1016 |
| macrolide antibiotic | 0.0953 | 0.0956 | 0.0954 | 0.0960 | 0.0963 | 0.0968 | 0.0945 | 0.0976 | 0.0970 | 0.0955 | 0.0947 | 0.0952 |
| fluoroquinolone antibiotic | 0.0831 | 0.0831 | 0.0826 | 0.0830 | 0.0809 | 0.0826 | 0.0802 | 0.0804 | 0.0806 | 0.0787 | 0.0788 | 0.0808 |
| peptide antibiotic | 0.0769 | 0.0776 | 0.0777 | 0.0753 | 0.0778 | 0.0758 | 0.0694 | 0.0713 | 0.0694 | 0.0674 | 0.0690 | 0.0677 |
| penam | 0.0717 | 0.0711 | 0.0711 | 0.0712 | 0.0706 | 0.0694 | 0.0746 | 0.0737 | 0.0746 | 0.0761 | 0.0755 | 0.0735 |
| disinfecting agents and antiseptics | 0.0602 | 0.0593 | 0.0599 | 0.0682 | 0.0667 | 0.0683 | 0.0747 | 0.0741 | 0.0743 | 0.0708 | 0.0725 | 0.0740 |
| cephalosporin | 0.0475 | 0.0478 | 0.0478 | 0.0480 | 0.0477 | 0.0479 | 0.0492 | 0.0477 | 0.0495 | 0.0509 | 0.0489 | 0.0498 |
| aminoglycoside antibiotic | 0.0419 | 0.0420 | 0.0416 | 0.0431 | 0.0440 | 0.0438 | 0.0504 | 0.0505 | 0.0521 | 0.0499 | 0.0498 | 0.0513 |
| phenicol antibiotic | 0.0400 | 0.0397 | 0.0402 | 0.0432 | 0.0423 | 0.0430 | 0.0434 | 0.0420 | 0.0413 | 0.0397 | 0.0428 | 0.0412 |
| carbapenem | 0.0390 | 0.0388 | 0.0390 | 0.0388 | 0.0379 | 0.0375 | 0.0404 | 0.0383 | 0.0395 | 0.0416 | 0.0407 | 0.0403 |
| cephamycin | 0.0362 | 0.0363 | 0.0365 | 0.0370 | 0.0355 | 0.0356 | 0.0382 | 0.0359 | 0.0379 | 0.0408 | 0.0386 | 0.0381 |
| glycopeptide antibiotic | 0.0312 | 0.0312 | 0.0311 | 0.0269 | 0.0268 | 0.0279 | 0.0228 | 0.0224 | 0.0234 | 0.0227 | 0.0236 | 0.0227 |
| monobactam | 0.0310 | 0.0313 | 0.0307 | 0.0297 | 0.0296 | 0.0289 | 0.0278 | 0.0251 | 0.0269 | 0.0297 | 0.0274 | 0.0275 |
| aminocoumarin antibiotic | 0.0282 | 0.0288 | 0.0285 | 0.0304 | 0.0313 | 0.0300 | 0.0280 | 0.0289 | 0.0300 | 0.0271 | 0.0278 | 0.0278 |
| rifamycin antibiotic | 0.0266 | 0.0270 | 0.0264 | 0.0235 | 0.0230 | 0.0244 | 0.0219 | 0.0224 | 0.0224 | 0.0219 | 0.0233 | 0.0229 |
| oxazolidinone antibiotic | 0.0177 | 0.0178 | 0.0174 | 0.0198 | 0.0194 | 0.0205 | 0.0248 | 0.0257 | 0.0242 | 0.0246 | 0.0258 | 0.0267 |
| penem | 0.0156 | 0.0152 | 0.0154 | 0.0154 | 0.0139 | 0.0137 | 0.0147 | 0.0124 | 0.0133 | 0.0166 | 0.0142 | 0.0139 |
| diaminopyrimidine antibiotic | 0.0149 | 0.0150 | 0.0151 | 0.0194 | 0.0187 | 0.0191 | 0.0195 | 0.0189 | 0.0176 | 0.0174 | 0.0182 | 0.0172 |
| lincosamide antibiotic | 0.0143 | 0.0144 | 0.0147 | 0.0121 | 0.0127 | 0.0122 | 0.0112 | 0.0126 | 0.0116 | 0.0128 | 0.0130 | 0.0140 |
| glycylcycline | 0.0135 | 0.0133 | 0.0133 | 0.0127 | 0.0124 | 0.0125 | 0.0102 | 0.0104 | 0.0104 | 0.0092 | 0.0099 | 0.0098 |
| mupirocin-like antibiotic | 0.0132 | 0.0136 | 0.0140 | 0.0101 | 0.0104 | 0.0110 | 0.0087 | 0.0088 | 0.0084 | 0.0089 | 0.0084 | 0.0091 |
| isoniazid-like antibiotic | 0.0129 | 0.0130 | 0.0128 | 0.0164 | 0.0179 | 0.0170 | 0.0156 | 0.0172 | 0.0156 | 0.0153 | 0.0150 | 0.0153 |
| fusidane antibiotic | 0.0115 | 0.0116 | 0.0115 | 0.0099 | 0.0098 | 0.0098 | 0.0070 | 0.0072 | 0.0076 | 0.0076 | 0.0069 | 0.0076 |
| phosphonic acid antibiotic | 0.0105 | 0.0105 | 0.0105 | 0.0097 | 0.0096 | 0.0096 | 0.0093 | 0.0089 | 0.0092 | 0.0096 | 0.0102 | 0.0094 |
| pleuromutilin antibiotic | 0.0102 | 0.0099 | 0.0102 | 0.0085 | 0.0086 | 0.0083 | 0.0079 | 0.0080 | 0.0078 | 0.0075 | 0.0079 | 0.0075 |
| streptogramin antibiotic | 0.0095 | 0.0094 | 0.0098 | 0.0074 | 0.0078 | 0.0079 | 0.0071 | 0.0083 | 0.0079 | 0.0080 | 0.0076 | 0.0080 |
| elfamycin antibiotic | 0.0071 | 0.0069 | 0.0069 | 0.0067 | 0.0070 | 0.0068 | 0.0063 | 0.0064 | 0.0070 | 0.0077 | 0.0064 | 0.0065 |
| pyrazine antibiotic | 0.0065 | 0.0064 | 0.0062 | 0.0059 | 0.0059 | 0.0062 | 0.0051 | 0.0048 | 0.0050 | 0.0055 | 0.0051 | 0.0054 |
| sulfonamide antibiotic | 0.0064 | 0.0063 | 0.0062 | 0.0067 | 0.0067 | 0.0063 | 0.0058 | 0.0052 | 0.0057 | 0.0054 | 0.0057 | 0.0055 |
| nybomycin-like antibiotic | 0.0061 | 0.0063 | 0.0065 | 0.0057 | 0.0055 | 0.0058 | 0.0040 | 0.0043 | 0.0041 | 0.0041 | 0.0038 | 0.0040 |
| nitroimidazole antibiotic | 0.0059 | 0.0059 | 0.0061 | 0.0048 | 0.0049 | 0.0052 | 0.0047 | 0.0046 | 0.0047 | 0.0054 | 0.0057 | 0.0050 |
| streptogramin A antibiotic | 0.0043 | 0.0044 | 0.0046 | 0.0038 | 0.0040 | 0.0043 | 0.0042 | 0.0046 | 0.0047 | 0.0050 | 0.0043 | 0.0046 |
| salicylic acid antibiotic | 0.0040 | 0.0040 | 0.0041 | 0.0038 | 0.0034 | 0.0039 | 0.0030 | 0.0031 | 0.0031 | 0.0031 | 0.0036 | 0.0031 |
| streptogramin B antibiotic | 0.0022 | 0.0021 | 0.0022 | 0.0017 | 0.0019 | 0.0021 | 0.0025 | 0.0029 | 0.0027 | 0.0038 | 0.0027 | 0.0031 |
| sulfone antibiotic | 0.0016 | 0.0014 | 0.0015 | 0.0014 | 0.0016 | 0.0014 | 0.0012 | 0.0012 | 0.0014 | 0.0013 | 0.0012 | 0.0013 |
| nucleoside antibiotic | 0.0015 | 0.0015 | 0.0017 | 0.0016 | 0.0014 | 0.0018 | 0.0024 | 0.0019 | 0.0020 | 0.0018 | 0.0017 | 0.0017 |
| nitrofuran antibiotic | 0.0014 | 0.0013 | 0.0013 | 0.0016 | 0.0016 | 0.0017 | 0.0009 | 0.0014 | 0.0011 | 0.0011 | 0.0011 | 0.0009 |
| bicyclomycin-like antibiotic | 0.0013 | 0.0014 | 0.0015 | 0.0015 | 0.0014 | 0.0015 | 0.0025 | 0.0022 | 0.0023 | 0.0023 | 0.0022 | 0.0022 |
| antibacterial free fatty acids | 0.0010 | 0.0010 | 0.0011 | 0.0011 | 0.0012 | 0.0012 | 0.0018 | 0.0017 | 0.0017 | 0.0016 | 0.0019 | 0.0015 |
| polyamine antibiotic | 0.0009 | 0.0007 | 0.0008 | 0.0013 | 0.0024 | 0.0012 | 0.0012 | 0.0014 | 0.0012 | 0.0009 | 0.0012 | 0.0009 |
| thioamide antibiotic | 0.0005 | 0.0005 | 0.0006 | 0.0011 | 0.0013 | 0.0011 | 0.0013 | 0.0015 | 0.0013 | 0.0013 | 0.0011 | 0.0012 |
| zoliflodacin-like antibiotic | 0.0005 | 0.0005 | 0.0006 | 0.0002 | 0.0002 | 0.0003 | 0.0003 | 0.0002 | 0.0003 | 0.0002 | 0.0002 | 0.0002 |
| orthosomycin antibiotic | 0.0001 | 0.0001 | 0.0001 | 0.0001 | 0.0001 | 0.0001 | 0.0001 | 0.0001 | 0.0001 | 0.0000 | 0.0001 | 0.0000 |

**
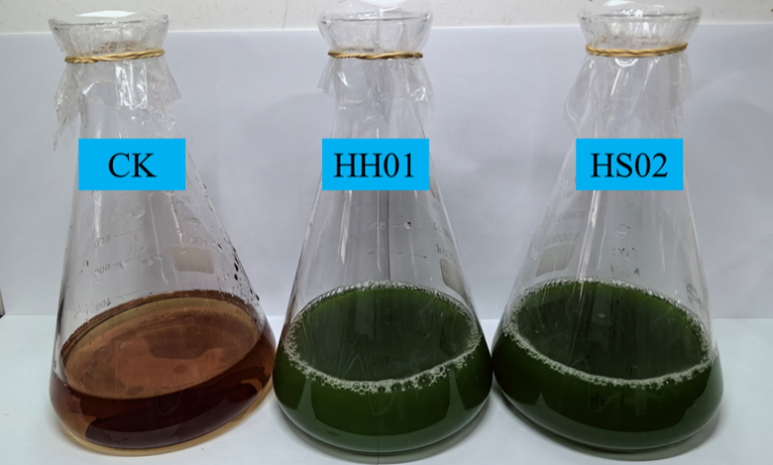
**

Fig. S1 Images of the experiment using microalgae to remediate a non-sterile biogas slurry after eight days of treatment


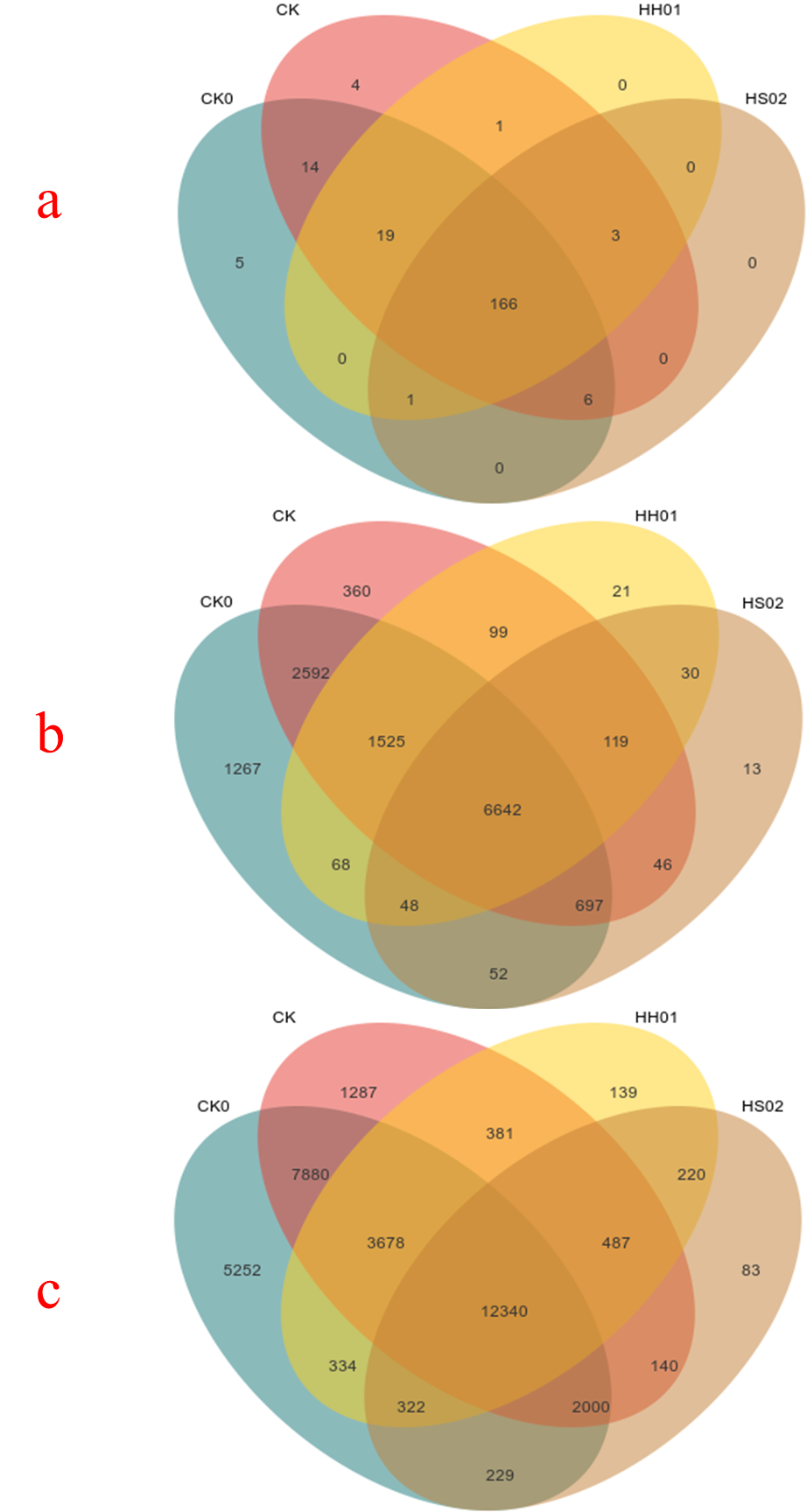


Fig. S2 Venn diagram of species differences (a, b, and c represent phylum, genus, and species level, respectively)
